# Kostenvergleich der konservativen vs. operativen Therapie des chronischen Lymphödems

**DOI:** 10.1007/s00104-024-02123-9

**Published:** 2024-06-28

**Authors:** Rima Nuwayhid, Stefan Langer, Nikolaus von Dercks

**Affiliations:** 1 Klinik für Orthopädie, Unfallchirurgie und Plastische Chirurgie, Universitätsklinikum Leipzig AöR, Liebigstraße 20, 04103 Leipzig, Deutschland; 2 Bereich Medizinmanagement, Universitätsklinikum Leipzig AöR, Liebigstraße 18, 04103 Leipzig, Deutschland

**Keywords:** Entstauungstherapie, Lymphknotentransfer, Liposuktion, Lymphovenöse Anastomose, Fallpauschale, Decongestive treatment, Lymph node transplantation, Liposuction, Lymphovenous anastomosis, Diagnosis related groups

## Abstract

**Hintergrund:**

Die Therapie des Lymphödems erfolgt primär konservativ mittels komplexer physikalischer Entstauungstherapie (KPE). Lymphovenöse Anastomosen (LVA), vaskularisierte Lymphknotentransplantationen (VLNT) und Liposuktionen stehen als operative Therapieverfahren zur Verfügung. Die Vergütung im DRG(„diagnosis related groups“)-System ist jedoch teils unzureichend oder nur nach individuellem Kostenübernahmeantrag möglich. Dabei sind die Kosten der verhältnismäßig neuen operativen Verfahren noch nicht in Relation zu denen der KPE gesetzt worden.

**Methodik:**

Die Kosten der leitliniengemäßen konservativen Therapie wurden ermittelt. Die Kosten für LVA, VLNT und Liposuktion jeweils an oberer und unterer Extremität wurden anhand der DRG-Fallpauschalen sowie der nach aktuellem Kenntnisstand erwarteten Reduktion konservativer Maßnahmen geschätzt. Anschließend erfolgte ein Vergleich der jährlichen Therapiekosten.

**Ergebnisse:**

Die jährlichen Therapiekosten nach LVA und VLNT sind bereits im 2. postoperativen Jahr niedriger als bei konservativer Therapie allein. Die Liposuktion erreicht diesen Punkt im 6. (obere Extremität) bzw. 47. postoperativen Jahr (untere Extremität).

**Diskussion:**

Die Evidenz für die positiven Effekte der Lymphchirurgie ist noch begrenzt. Es ist jedoch erkennbar, dass der kurative operative Ansatz sowohl die Therapiekosten deutlich senken als auch die Lebensqualität Betroffener verbessern kann. Es mangelt jedoch an einer adäquaten Abbildung des operativen Aufwands in der Vergütung.

**Zusatzmaterial online:**

Zusätzliche Informationen sind in der Online-Version dieses Artikels (10.1007/s00104-024-02123-9) enthalten.

## Abstract (en)

**Background:**

Lymphedema is primarily treated conservatively using complex physical decongestion treatment (CDT). Lymphovenous anastomosis (LVA), vascularized lymph node transplantation (VLNT) and liposuction are available as surgical treatment methods; however, reimbursement in the diagnosis-related groups (DRG) system is sometimes inadequate or only possible following an individual application. The costs of these relatively new surgical procedures have not yet been set in relation to those of CDT.

**Method:**

The costs of conservative treatment were determined in accordance with the guidelines. The costs for LVA, VLNT and liposuction of the upper and lower extremities were estimated on the basis of the DRG reimbursement per case and the expected reduction in conservative measures according to current knowledge. The annual treatment costs were then compared.

**Results:**

The annual treatment costs of LVA and VLNT are already lower than conservative treatment alone in the second postoperative year. Liposuction reaches this point in the 6th (upper extremity) or 47th postoperative year (lower extremity).

**Conclusion:**

The evidence for the positive effects of lymphatic surgery is still limited; however, it is recognizable that the curative surgical approach can significantly reduce the treatment costs and improve the quality of life of lymphedema patients; however, there is a lack of adequate reflection of the surgical effort in the reimbursement.

Patient:innen mit Lymphödem steht auch die Option verschiedener chirurgischer Therapieverfahren zur Verfügung. Während die Effektivität der Verfahren zunehmend belegt wird, stellt die Kostenübernahme nach wie vor eine Hürde für Patient:innen und behandelnde Chirurg:innen dar. Die Vergütung für Lymphknotentransplantationen ist undefiniert und unzureichend, die Kosten für Liposuktionen werden nur nach individuellen Kostenübernahmeanträgen genehmigt. In diesem Beitrag sollen die gesundheitsökonomischen Kosten der konservativen Therapie denen der operativen Verfahren gegenübergestellt werden.

## Hintergrund

Lymphödeme resultieren aus einem Ungleichgewicht von anfallender lymphpflichtiger Last und der Transportkapazität des Lymphgefäßsystems. Klinisch präsentiert sich das Lymphödem als Schwellung der betroffenen Areale, meist der Extremitäten. Es werden primäre von sekundären Lymphödemen unterschieden. Primäre Lymphödeme sind mit einer Prävalenz von 1:100.000 als selten zu betrachten [9]. Während weltweit gesehen die Filariose, eine parasitäre Erkrankung hervorgerufen durch *Wucheria bancrofti,* die häufigste Ursache für Lymphödeme ist, sind sie in den Industrienationen meist die Folge einer Krebserkrankung und deren Behandlung [9].

Die Gültigkeit der deutschsprachigen S2k-Leitlinie *Diagnostik und Therapie der Lymphödeme* ist 2022 abgelaufen [8]. Grundlegende Änderungen in Bezug auf die hier untersuchten Empfehlungen sind bei weitgehender Übereinstimmung mit denen der International Society of Lymphology sowie anderen nationalen Leitlinien jedoch auch in der in Erarbeitung befindlichen S3-Leitlinie nicht zu erwarten [21, 22].

### Konservative Therapie

Primär erfolgt die Therapie des Lymphödems konservativ durch mehrere Komponenten, zusammengefasst als komplexe physikalische Entstauungstherapie (KPE). Unter diesen multidisziplinären Therapiekomplex fallen Hautpflege, manuelle Lymphdrainage (MLD), Kompressionstherapie, entstauungsfördernde Bewegungstherapie sowie die Schulung zur Selbsttherapie [8].

#### Manuelle Lymphdrainage

Die manuelle Lymphdrainage hat die Mobilisierung der im Interstitium akkumulierten Flüssigkeit zum Ziel und wird klassifiziert als Massagetherapie gemäß § 18 der Heilmittelrichtlinie. Nach Aufnahme des Lymphödems ab Stadium 2 in die Diagnosenliste für den langfristigen Heilmittelbedarf erfolgt die Verordnung außerhalb des Regelfalles.

Die Heilmittelverordnung sieht die Verordnung von Lymphdrainage für 30, 45 oder 60 min ein- bis dreimal wöchentlich vor [16]. Zur Kontrolle des Therapieerfolgs sowie zur erneuten Verordnung sind laut § 7 (6) Heilmittelverordnung ärztliche Vorstellungen alle 12 Wochen notwendig [16].

#### Hilfsmittel zur Kompressionstherapie

Essenzieller Bestandteil der KPE ist die Kompressionstherapie. Durch das tägliche Tragen medizinischer Kompressionswäsche soll u. a. einer erneuten Akkumulation interstitieller Flüssigkeit nach deren erfolgreicher Mobilisierung vorgebeugt werden. Bedingt durch eine nachlassende Elastizität der Fasern werden zwei Versorgungen pro Jahr verordnet [8]. Da die Kompressionswäsche nur über Nacht nicht getragen wird, besteht ein kurzes Zeitfenster für die täglich notwendige Reinigung und Trocknung der Kompressionswäsche, sodass auch eine hygienische Wechselversorgung notwendig ist. Hieraus ergeben sich in der Regel vier Verordnungen von Kompressionswäsche pro Patient und Jahr.

#### Erysipeltherapie

Das Ödem bedingt eine Störung der Lymphozytenzirkulation, hierdurch ist das Auftreten von Infektionen begünstigt. Rezidivierende Erysipele sind daher häufige Komplikationen des Lymphödems. Lymphödempatient:innen erleiden 0,25 Episoden pro Jahr [20].

Laut der S2k-Leitlinie *Kalkulierte parenterale Initialtherapie bakterieller Erkrankungen bei Erwachsenen* (Status: in Überarbeitung) besteht bei „z. B. venöse[r] oder arterielle[r] Durchblutungsstörung“ ein kompliziertes Erysipel, bei welchem die parenterale und somit im Regelfall stationäre antimikrobielle Therapie indiziert ist.

### Operative Therapie

Zahlreiche Studien zum Effekt lymphchirurgischer Eingriffe sind mittlerweile publiziert, die Ergebnisse sind jedoch aufgrund variierender Methodik in Bezug auf die operative Technik als auch die Wahl der Outcomepprameter nur eingeschränkt vergleichbar. Aus der vorliegenden Literatur lässt sich dennoch ableiten, dass sowohl die lymphovenöse Anastomose (LVA), die vaskularisierte Lymphknotentransplantation („vascularised lymph node transplant/transfer“, VLNT) als auch die Liposuktion eine Reduktion der Umfänge der betroffenen Extremitäten sowie der Erysipelraten bewirken und teilweise die Notwendigkeit des Tragens von Kompressionsbekleidung aufheben kann [6, 10, 14, 19, 23–25]. Alle drei Verfahren gelten als vergleichsweise sicher [10, 17, 23, 24].

#### Lymphovenöse Anastomose

Für eine lymphovenöse Anastomose erfolgt im Rahmen eines supramikrochirurgischen Eingriffs der Anschluss peripherer Lymphgefäße an das venöse System, damit wird ein sofortiger Lymphabfluss ermöglicht.

#### Vaskularisierte Lymphknotentransplantation

Die VLNT entspricht einer autologen Transplantation lymphknotenhaltigen Gewebes. Verschiedene Donorareale sind beschrieben [3]. In der vorliegenden Untersuchung beschränken wir uns auf die von uns präferierte laparoskopische Teilomentektomie zur Transplantatgewinnung.

Sowohl LVA als auch VLNT reduzieren an OE und UE mit dem Extremitätenumfang auch die Rate an auftretenden Erysipelen sowie die Notwendigkeit des Tragens von Kompressionskleidung [1, 10, 23, 25].

#### Liposuktion

Die Liposuktion reduziert das subkutane Volumen der Extremität sofort und anhaltend, allein angewendet therapiert sie jedoch die zugrunde liegende Störung des lymphatischen Abflusses nicht [7]. Daher ist nach Liposuktion allein weiterhin MLD und das Tragen von Kompressionswäsche notwendig. Die Liposuktionsbehandlung allein kann jedoch eine deutliche Reduktion der Erysipelrate erreichen [12, 13]. Sie hat zudem den größten Einfluss auf die Angleichung der Asymmetrie der Extremitätenumfänge, da sie als einzige Methode die aus der interstitiellen Inflammation resultierende Fettgewebshypertrophie direkt adressiert [2].

## Methodik

### Errechnete Einschätzung der Kosten der konservativen Therapie

#### Manuelle Lymphdrainage

Wir gehen für unsere Berechnungen von zweimal wöchentlich 45 min aus und dies in 46 Wochen pro Jahr, welche sich aus dem Abzug von durchschnittlich 30 Tagen Jahresurlaub ergeben. Hinzu kommt die EBM(Einheitlicher Bewertungsmaßstab)-Kalkulation für die vorgesehene quartalsweise ärztliche Vorstellung zur Kontrolle und Weiterverordnung.

#### Hilfsmittel zur Kompressionstherapie

Für Lymphödeme der OE wird ein Kompressionsoberarmstrumpf mit einem Handschuh verordnet, für Lymphödeme der UE ein Paar Oberschenkelstrümpfe, jeweils nach Maß flachgestrickt. Wir gingen bei zwei Verordnungen pro Jahr mit jeweils einer hygienischen Wechselversorgung von insgesamt vier Versorgungen pro Jahr aus.

#### Erysipeltherapie

Zur Kalkulation zogen wir die DRG J64B ohne Aufschläge für die stationäre Behandlung von Komplikationen heran.

### Errechnete Einschätzung der Kosten der operativen Therapie

Für die vorliegende Modellierung nehmen wir an, dass die Kosten der konservativen Therapie im Jahr der Operation so hoch sind wie unter konservativer Therapie allein. Ab dem 1. Jahr nach der Operation wurden dann die Kosten der konservativen Therapie nur noch anteilig berechnet entsprechend der Reduktion, welche im Zusammenhang mit der jeweiligen Operationsmethode in der Literatur berichtet wird (siehe Tab. 1 und 2 im Onlinematerial). In der KPE folgt auf MLD stets Kompressionsbehandlung, um die durch Mobilisierung der interstitiellen Flüssigkeit erreichte Volumenreduktion zu konservieren. Wir gingen daher davon aus, dass der Verzicht auf das Tragen von Kompressionskleidung auch den Verzicht auf MLD bedeutet (Tab. 1).

**Tab. 1:** Darstellung der Reduktion der Notwendigkeit des Tragens von Kompressionskleidung sowie der Erysipelfrequenz in Abhängigkeit von der Operationsmethode

	Verzicht auf Kompressionskleidung	Reduktion der jährlichen Erysipelrate
*Obere Extremität*		
LVA	66 % [10]	93 % [10]
VLNT	51 % [25]	73 % [25]
Liposuktion	–	95 % [12]
*Untere Extremität*		
LVA	56 % [1]	92 % [23]
VLNT	78 % [1]	71 % [5]
Liposuktion	–	87 % [10, 13]

*LVA* lymphovenöse Anastomosen, *VLNT* vaskularisierte Lymphknotentransplantationen

#### LVA

Die LVA wird stationär erbracht und bei Behandlung eines Lymphödems der oberen Extremität (z. B. nach Mammaeingriff) mit der DRG J07A vergütet. Das entspricht einem Erlös von 5917,50 €. Bei Lymphödem der unteren Extremitäten (z. B. nach Zervixkarzinom) wird die DRG 801E mit einem Erlös in Höhe von 7953,09 € angesteuert.

#### VLNT

Die VLNT wird stationär erbracht und führt bei Behandlung eines Lymphödems der oberen Extremität ebenfalls in die DRG J07A mit gleichem Erlös wie bei der LVA. Bei Lymphödem der unteren Extremitäten erfolgt die stationäre Behandlung über die DRG 802A mit einem Erlös in Höhe von 9349,40 €.

#### Liposuktion

Bei der Liposuktion aufgrund eines Lymphödems erfolgt die Vergütung der stationären Behandlung an der oberen Extremität über die DRG J10B (2628,60 €), bei Behandlung an der unteren Extremität über die DRG K09D (3709,48 €). Die Angaben der Erlöse aus den Fallpauschalen berücksichtigen keine Zu- oder Abschläge und enthalten keine Pflegeerlöse, da diese über das Pflegebudget finanziert sind.

## Ergebnisse

Für die rein konservative Therapie eines Lymphödems der OE ergeben sich jährliche Kosten von schätzungsweise 7218 € mit nur geringem Unterschied zur UE mit 7597 €. Im Jahr der Operation entstehen für die Durchführung von LVA an der OE samt begleitender konservativer Therapie Kosten von 13.135 €, ab Eintreten der berichteten Effekte im 1. postoperativen Jahr reduziert dieser Betrag sich auf 2310 € jährlich. An der unteren UE entstehen durch LVA zunächst 15.551 € Kosten mit Reduktion auf jährlich 3149 €.

Die VLNT an der OE kostet im Operationsjahr 13.135 € und in der Folge 2310 €. An der UE entstehen im Jahr des Eingriffs Kosten in Höhe von 16.947 € mit Reduktion auf 1709 € nach Eintritt des Effekts.

Da die Liposuktion keinen Einfluss auf das Tragen von Kompressionskleidung und MLD hat, wirkt sie sich nur im Bereich der Erysipeltherapie kostensenkend aus. Im Jahr der Operation entstehen noch Kosten von 9847 € an der OE und 11.307 € an der UE, welche sich auf 6707 € bzw. 7130 € reduzieren. Eine tabellarische Aufstellung findet sich in den Begleitmaterialien online.

**Abb. 1:**
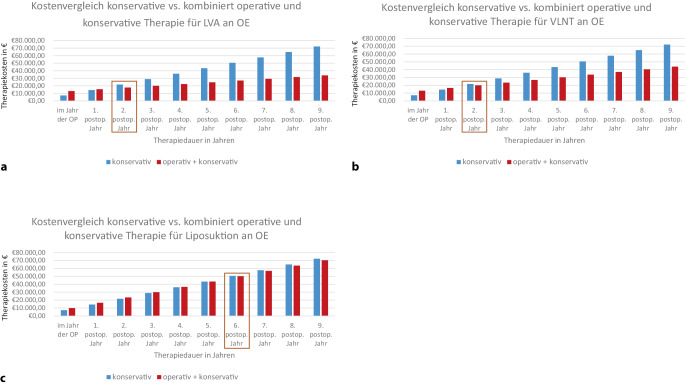
Grafische Darstellung der jährlichen Therapiekosten bei konservativer (*blau*) und kombiniert operativer und konservativer (*rot*) Therapie des chronischen Lymphödems der oberen Extremität (*OE*). Therapiekosten **a** bei lymphovenöser Anastomose (*LVA*), **b** bei vaskularisierter Lymphknotentransplantation (*VLNT*) und **c** bei Liposuktion. *Kasten*: Break-even-Point, ab welchem die Kosten der konservativen Therapie die der kombiniert operativen und konservativen übersteigen. *OP* Operation

Somit reduzieren sich die geschätzten jährlichen Behandlungskosten mit jeder der drei Operationsmethoden. Der Zeitpunkt, ab welchem die zusätzlichen Kosten der Operation durch die Reduktion konservativer Therapie amortisiert sind, ist in den Abb. 1 und 2 grafisch dargestellt.

**Abb. 2:**
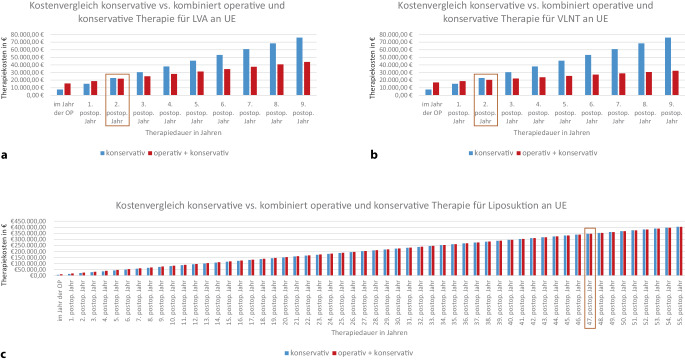
Grafische Darstellung der jährlichen Therapiekosten bei konservativer (*blau*) und kombiniert operativer und konservativer (*rot*) Therapie des chronischen Lymphödems der unteren Extremität (*UE*). Therapiekosten **a** bei lymphovenöser Anastomose (*LVA*), **b** bei vaskularisierter Lymphknotentransplantation (*VLNT*) und **c** bei Liposuktion. *Kasten*: Break-even-Point, ab welchem die Kosten der konservativen Therapie die der kombiniert operativen und konservativen übersteigen. *OP* Operation

## Diskussion

In der vorliegenden Arbeit untersuchten wir die geschätzten Kosten der primär konservativen Therapie des chronischen Lymphödems und verglichen diese mit den Kosten verschiedener Verfahren der operativen Therapie unter Berücksichtigung zu erreichender Effekte. Die Berechnungen zeigten, dass alle drei operativen Verfahren sowohl an der OE als auch UE zu einer Reduktion der Gesundheitskosten führen. Die Therapiekosten für LVA und VLNT liegen bereits im 2. postoperativen Jahr unter denen der rein konservativen Therapie.

Die Kosten der operativen Therapie wurden nicht isoliert betrachtet, da keine Operationsmethode mit hundertprozentiger Rate die konservative Therapie überflüssig macht. Es handelt sich also langfristig um eine Kombination aus operativer und reduzierter konservativer Therapie.

### Limitationen

Das Ziel der vorliegenden Arbeit war eine Gegenüberstellung der multifaktoriell entstehenden Kosten. Hierzu griffen wir auf international publizierte Daten zu den Effekten lymphchirurgischer Eingriffe zurück. Die Proband:innenzahlen in diesen Studien sind jedoch vergleichsweise gering, selbst Metaanalysen konnten nur Daten zu zweistelligen bis niedrig dreistelligen Patient:innenzahlen erfassen. Stark differierende Darstellungen der Outcomeparameter erschwerten hier eine Vereinheitlichung. Dennoch wurden diese Zahlen bereits für eine Kosten-Nutzen-Analyse zwischen LVA und VLNT herangezogen [18].

Des Weiteren betragen die Nachbeobachtungszeiträume der Studien maximal wenige Jahre, sodass unklar ist, ob die beschriebenen Effekte tatsächlich ein Leben lang aufrechterhalten werden können.

Für unsere Berechnungen gingen wir von einer Verbesserung der Beschwerden ein Jahr postoperativ aus. Dies entspricht sowohl unserer eigenen als auch der Erfahrung anderer Operateure [4, 6]. Selbst wenn wir jedoch davon ausgehen, dass in den ersten 5 Jahren ab der Operation keinerlei Effekt eintritt, ist der Break-even-Point beispielhaft für die LVA an der OE dennoch bereits im 7. postoperativen Jahr erreicht.

Betrachtet wurde hier die einmalige Operation eines unilateralen Lymphödems, was dem Regelfall in den Industrienationen entspricht. Doch auch im Falle einer bilateralen oder wiederholten Operation wird der Break-even-Punkt noch im Laufe der Lebenserwartung erreicht.

In Studien zu lymphchirurgischen Eingriffen werden als Komplikationen beinahe ausschließlich Minorkomplikationen angegeben, am häufigsten Serome und Wundinfektionen sowohl im Donor- als auch Akzeptorareal [10, 17, 23, 24]. Da diese Komplikationen zumeist wenige Tage postoperativ auftreten, fallen sie noch in den stationären Aufenthalt, sodass ihre Behandlung bereits durch den DRG-Erlös abgegolten ist. Die Differenzen in den jährlichen Therapiekosten sind so groß, dass selbst bei zusätzlicher Kalkulation einer stationären Therapie eines Erysipels die Kosten immer noch bereits im 2. postoperativen Jahr niedriger wären als bei konservativer Therapie allein.

### Moderne Therapieverfahren brauchen eine moderne Abrechnung

Die VLNT ist im deutschen DRG-System nicht adäquat abgebildet. Das liegt maßgeblich am Fehlen einer adäquaten Kodierbarkeit der Prozeduren. Die Entnahme der Lymphknoten erfolgt beim von uns favorisierten Vorgehen aus dem Omentum majus, das hierfür laparoskopisch partiell reseziert wird. Das ist in dieser Form nicht im Operationen- und Prozedurenschlüssel (OPS) abgebildet. Somit sind nur die „sonstige“ Lymphgefäßentnahme und die entsprechende Transplantation zu kodieren. Klassifikatorisch wäre hier eine differenzierende OPS-Kodierung als Kostentrenner erforderlich. Die behelfsweise Kodierung als Lappenplastik ist nicht zulässig, da gefäßgestielte reine Fettgewebslappen nicht kodierbar sind. Diese Problematik besteht auch für Transplantate anderer Donorareale, die ohne Hautinsel entnommen werden.

Angestrebtes Vorgehen durch uns ist die Beantragung eines spezifischen OPS-Codes für die VLNT. Damit kann für das Institut für das Entgeltsystem im Krankenhaus (InEK) künftig eine Kalkulationsgrundlage dieser aufwendigen Eingriffe geschaffen werden.

### Unzureichende Evidenzlage für konservative Therapie

Die Evidenzlage für die Wirkung der konservativen Therapie ist unzureichend [7]. Weder für Kompressionstherapie noch MLD als Monotherapie existieren kontrollierte Studien mit dem Nachweis eines hinreichenden Effekts bei bereits bestehendem Lymphöden. Insbesondere die ressourcenintensive MLD konnte in einer Metaanalyse keine signifikante Volumenreduktion über den Effekt der Kompressionstherapie hinaus bewirken [11]. Eine positive Beeinflussung durch eine Kombinationstherapie, wie bei der KPE intendiert, ist möglich und zeigte sich auch in der einzigen vergleichenden Studie zwischen VLNT und KPE [6]. Hier war die KPE der VLNT jedoch in allen untersuchten Aspekten signifikant unterlegen.

### Liposuktion als sinnvolle therapeutische Ergänzung

Mit zeitlichem Abstand ist auch die Kombination mit einer Liposuktion sinnvoll. Die Liposuktion ist keine kurative Therapie, sie kann jedoch die Bedingungen für den Lymphabfluss verbessern und daher eine sinnvolle supportive Maßnahme für lymphrekonstruktive Eingriffe sein. Durch die deutliche Reduktion des Auftretens von Erysipelen wird zudem der Verbrauch von Antibiotika gesenkt. Die Liposuktion verhilft den Patient:innen am schnellsten und effektivsten zu einer Reduktion des Extremitätenumfangs, da die beiden deviierenden Verfahren LVA und VLNT keine Reduktion des hypertrophierten subkutanen Fettgewebes erreichen können.

Wie die positiven Effekte sich bei Kombination verschiedener Verfahren entwickeln, ist unklar und konnte deshalb nicht in die Berechnungen einfließen. Selbst wenn wir jedoch davon ausgingen, dass die zusätzliche Liposuktion keinerlei positiven Effekt auf die Erysipelrate hat als der deviierende Eingriff allein und beispielhaft die Kalkulation für LVA und Liposuktion an der UE in getrennten Fällen durchführen, sind die Therapiekosten ab dem 5. postoperativen Jahr geringer gegenüber der rein konservativen Therapie.

Wenn die im Verhältnis deutlich höheren Kosten für die empirische und rein symptomatische konservative Therapie trotz der unzureichenden Evidenzlage übernommen werden, wäre es auch in Anbetracht der verhältnismäßig geringen Komplikationsraten wünschenswert, dass die kurative operative Therapie ausreichend vergütet wird und nach fachärztlicher Indikationsstellung ohne Beantragung einer Kostenübernahme durchgeführt werden kann [17].

### Nicht nur eine Frage des Geldes, sondern der Lebensqualität

Die Perspektive dieser Arbeit ist eine primär gesundheitsökonomische. Bei der Evaluation des Nutzens lymphchirurgischer Eingriffe ist jedoch nicht außer Acht zu lassen, dass alle hier genannten Methoden bei einer großen Mehrheit der Patient:innen zu einer subjektiven Befundbesserung führen und einen positiven Effekt auf ihre Lebensqualität haben, da insbesondere die aufwendige konservative Therapie eine relevante Einschränkung darstellt [1, 10, 15, 23–25].

Zusammenfassend wird auch unter Berücksichtigung der Limitationen dieser kursorischen Berechnungen deutlich, dass in Anbetracht der chronisch-progredienten Natur des Lymphödems keine ökonomischen Gründe gegen die operativen Therapiemethoden sprechen. Dies ist insbesondere den hohen Kosten der konservativen Maßnahmen geschuldet, welche zudem als rein symptomatische Therapie lebenslang durchgeführt werden müssen.

## Fazit für die Praxis

- Die Therapie des Lymphödems erfolgt primär konservativ mittels komplexer physikalischer Enstauungstherapie (KPE).
- KPE ist jedoch ressourcenintensiv, belastend für die Patient:innen und zudem lebenslang notwendig.
- Lymphovenöse Anastomose (LVA), vaskularisierte Lymphknotentransplantation/-transfer (VLNT) und Liposuktion reduzieren die Notwendigkeit konservativer Therapiemaßnahmen und damit die Therapiekosten bei Patient:innen mit chronischem Lymphödem der oberen (OE) oder unteren Extremität (UE).
- Bereits im 2. postoperativen Jahr ist die Lymphödemtherapie nach LVA oder VLNT an der OE oder UE wirtschaftlicher als die konservative Therapie.
- Die Vergütung im DRG(„diagnosis related groups“)-System ist dem operativen Aufwand jedoch nicht angemessen.
- Die Liposuktion ist eine sinnvolle, ergänzende operative Maßnahme, deren Kostenübernahme aktuell noch gesondert beantragt werden muss.

## Supplementary Information

Tabelle 1: Kostenvergleich der konservativen versus kombiniert operativen und konservativen Therapie des chronischen Lymphödem der oberen Extremität; Tabelle 2: Kostenvergleich der konservativen versus kombiniert operativen und konservativen Therapie des chronischen Lymphödem der unteren Extremität

## Abkürzungen

DRG: Diagnosis related groups
KPE: Komplexe physikalische Enstauungstherapie
LVA: Lymphovenöse Anastomose
MLD: Manuelle Lymphdrainage
OE: Obere Extremität
OPS: Operationen- und Prozedurenschlüssel
UE: Untere Extremität
VLNT: Vaskularisierte Lymphknotentransplantation/-transfer
