# Supplementary material for: Cost comparison of conservative vs. surgical treatment of chronic lymphedema
Source: Chirurgie (Heidelb). 2024 Jun 28;96(1):41–7. [Article in German] doi: 10.1007/s00104-024-02123-9 (PMC11729079; doi:10.1007/s00104-024-02123-9)
Supplement: Supplementary file 1 — Tabelle 1: Kostenvergleich der konservativen versus kombiniert operativen und konservativen Therapie des chronischen Lymphödem der oberen Extremität; Tabelle 2: Kostenvergleich der konservativen versus kombiniert operativen und konservativen Therapie des chronischen Lymphödem der unteren Extremität [file 104_2024_2123_MOESM1_ESM.docx]

**Begleitmaterial zu**

**Kostenvergleich der konservativen versus operativen Therapie des chronischen Lymphödems**

Rima Nuwayhid, Stefan Langer, Nikolaus von Dercks

Tabelle 1: Vergleich der geschätzten durchschnittlichen jährlichen Behandlungskosten für die konservative versus kombiniert operative und konservative Therapie des chronischen Lymphödems der oberen Extremität.

| Posten | Kosten | Faktor | Jährliche Kosten der konservativen Therapie | Jährliche Kosten im Jahr der LVA | Jährliche Kosten ab dem ersten postoperativen Jahr der LVA | Jährliche Kosten im Jahr der VLNT | Jährliche Kosten ab dem ersten postoperativen Jahr der VLNT | Jährliche Kosten im Jahr der Liposuktion | Jährliche Kosten ab dem ersten postoperativen Jahr der VLNT |
| --- | --- | --- | --- | --- | --- | --- | --- | --- | --- |
| Lymphovenöse Anastomose  DRG J07A  OPS 5-408.0 | 5.917,50 € | / | / | 5.917,50 € | / | / | / | / | / |
| Lymphknotentransplantation von omental  DRG J07A  OPS 5-408.60, 5-401.fx | 5.917,50 € | / | / | / | / | 5.917,50 € | / | / | / |
| Liposuktion Ober- und Unterarm  DRG J10B  OPS 5-911.17 und 5-911.18 | 2628,60 € | / | / | / | / | / | / | 2.628,60 € | / |
| 1 Sitzung MLD à 45 min | 50,60 € | MLD 2x wöchentlich,  52 Jahreswochen – 6 Wochen Urlaub = 46 Wochen  → x 2 x 46 | 4.655,20 € | 4.655,20 € | 1.583,77 €  Reduktion um 66% | 4.655,20 € | 2.281,10 €  Reduktion um 51% | 4.655,20 € | 4.655,20 €  Kein Einfluss |
| Ambulante Arztbesuche | 61,10 EUR | Aller 12 Wochen zur Verordnung MLD  → x 4 | 244,40 € | 244,40 € | 83,10 €  Reduktion um 66% | 244,40 € | 119,76 €  Reduktion um 51% | 244,40 € | 244,40 €  Kein Einfluss |
| 1 Oberarmstrumpf nach Maß, flach gestrickt, Kompressionsklasse 2 | ca. 215 € | Verordnung 2 x jährlich + hygienische Wechselversorgung → x 4 | 860 € | 860 € | 292 €  Reduktion um 66% | 860 € | 421 €  Reduktion um 51% | 860 € | 860 €  Kein Einfluss |
| 1 Kompressionshandschuh nach Maß, flach gestrickt, Kompressionsklasse 2 | ca. 230 € | Verordnung 2 x jährlich + jeweils hygienische Wechselversorgung  → x 4 | 920 € | 920 € | 313 €  Reduktion um 66% | 920 € | 451 €  Reduktion um 51% | 920 € | 920 €  Kein Einfluss |
| Stationäre Therapie Erysipel | DRG J64B, 2153,35 € | 0,25 Erysipelepisoden jährlich  → x 0,25 | 538,34 € | 538,34 € | 37,68 €  Reduktion um 93% | 538,34 € | 145,35 €  Reduktion um 73% | 538,34 € | 26,92 €  Reduktion um 95% |
|  |  | **Gesamtkosten jährlich** | **7.217,94 €** | **13.135,44 €** | **2.309,55 €** | **13.135,44 €** | **3.418,21 €** | **9.846,54 €** | **6.706,52 €** |

Tabelle 2: Vergleich der geschätzten durchschnittlichen jährlichen Behandlungskosten für die konservative versus kombiniert operative und konservative Therapie des chronischen Lymphödems der unteren Extremität.

| Posten | Kosten | Faktor | Jährliche Kosten der konservativen Therapie | Jährliche Kosten im Jahr der LVA | Jährliche Kosten ab dem ersten postoperativen Jahr der LVA | Jährliche Kosten im Jahr der VLNT | Jährliche Kosten ab dem ersten postoperativen Jahr der VLNT | Jährliche Kosten im Jahr der Liposuktion | Jährliche Kosten ab dem ersten postoperativen Jahr der VLNT |
| --- | --- | --- | --- | --- | --- | --- | --- | --- | --- |
| Lymphovenöse Anastomose  DRG 801E  OPS 5-408.0 | 7.953,09 € | / | / | 7.953,09 € | / | / | / | / | / |
| Lymphknotentransplantation von omental  DRG 802A  OPS 5-408.61, 5-401.fx | 9.349,40 € | / | / | / | / | 9.349,40 € | / | / | / |
| Liposuktion Ober- und Unterschenkel  DRG K09D  OPS 5-911.1e und 5-911.1f | 3.709,48 € | / | / | / | / | / | / | 3.709,48 € | / |
| 1 Sitzung MLD à 45 min | 50,60 € | MLD 2x wöchentlich,  52 Jahreswochen – 6 Wochen Urlaub = 46 Wochen  → x 2 x 46 | 4.655,20 € | 4.655,20 € | 2.048,29 €  Reduktion um 56% | 4.655,20 € | 1.024,14 €  Reduktion um 78% | 4.655,20 € | 4.655,20 € |
| Ambulante Arztbesuche | 61,10 EUR | Aller 12 Wochen zur Verordnung MLD  → x 4 | 244,40 € | 244,40 € | 107,54 €  Reduktion um 56% | 244,40 € | 53,77 €  Reduktion um 78% | 244,40 € | 244,40 €  Kein Einfluss |
| 1 Paar Oberschenkelstrümpfe nach Maß, flach gestrickt, Kompressionsklasse 2 | ca. 540 € | Verordnung 2 x jährlich + hygienische Wechselversorgung → x 4 | 2.160 € | 2.160 € | 950,40 €  Reduktion um 56% | 2.160 € | 475,20 €  Reduktion um 78% | 2.160 € | 2.160 €  Kein Einfluss |
| Stationäre Therapie Erysipel | DRG J64B 2153,35 € | 0,25 Erysipelepisoden jährlich  → x 0,25 | 538,34 € | 538,34 € | 43,10 €  Reduktion um 92% | 538,34 € | 156,12 €  Reduktion um 71% | 538,34 € | 69,99 €  Reduktion um 87% |
|  |  | **Gesamtkosten jährlich** | **7.597,94 €** | **15.551,03 €** | **3.149,33 €** | **16.947,34 €** | **1.709,23 €** | **11.307,42 €** | **7.129,59 €** |

Tabelle 3: Angabe der jährlichen Therapiekosten der jeweiligen Operationsmethode zum Zeitpunkt des Break-even-Punktes, ab welchem die Kosten der kombiniert operativen und konservativen Therapie geringer sind als die der rein konservativen Therapie.

| **Operationsmethode** | **OE**  (konservativ vs. operativ + konservativ) | **UE**  (konservativ vs. operativ + konservativ) |
| --- | --- | --- |
| **LVA** | 2. postoperatives Jahr  (21.654 € vs. 17.755 €) | 2. postoperatives Jahr  (22.794 € vs. 21.850 €) |
| **VLNT** | 2. postoperatives Jahr  (21.654 € vs. 19.972 €) | 2. postoperatives Jahr  (22.794 € vs. 20.366 €) |
| **Liposuktion** | 6. postoperatives Jahr  (50.526 € vs. 50.086 €) | 47. postoperatives Jahr  (346.461 € vs. 346.398 €) |
